# Screening Depression in Ischemic Heart Disease: Gender Differences and Psychosocial Implications Using a Self-Developed Questionnaire

**DOI:** 10.3390/jcm14030837

**Published:** 2025-01-27

**Authors:** Laura Ioana Bondar, Diana Carina Iovanovici, Victor Măduța, Denis Bogdan Butari, Florin Mihai Șandor, Mariana Adelina Mariș, Ligia Elisaveta Piroș, Caius Calin Miuța, Corina Dalia Toderescu, Mircea Ioachim Popescu

**Affiliations:** 1 Doctoral School of Biomedical Sciences, University of Oradea, 410087 Oradea, Romania; bondar.lauraioana@student.uoradea.ro (L.I.B.); diana_iovanovici@yahoo.com (D.C.I.); procardia_oradea@yahoo.com (M.I.P.); 2 Department of Biology and Life Sciences, Faculty of Medicine, “Vasile Goldiș” Western University of Arad, 310048 Arad, Romania; maduta.victor@uvvg.ro (V.M.); butari.denis-bogdan@uvvg.ro (D.B.B.); sandor.florin@uvvg.ro (F.M.Ș.); 3 Department of General Medicine, Faculty of Medicine, “Vasile Goldiș” Western University of Arad, 310048 Arad, Romania; maris.mariana@uvvg.ro (M.A.M.); piros.ligia@uvvg.ro (L.E.P.); 4 Faculty of Physical Education and Sport, “Aurel Vlaicu” University of Arad, 310130 Arad, Romania; 5 Faculty of Medicine, “Victor Babeș” University of Medicine and Pharmacy, 300041 Timisoara, Romania; corina.toderescu@umft.ro

**Keywords:** depression screening, gender differences, ischemic heart disease, risk factors, self-report questionnaire, well-being

## Abstract

**Background/Objectives**: Ischemic heart disease (IHD) is a major cause of morbidity and mortality worldwide, and it is frequently associated with depression, which can negatively impact both clinical outcomes and quality of life. The relationship between IHD and depression is complex, with gender differences influencing the severity of depression and willingness to seek psychological support. This study aims to evaluate the prevalence and severity of depression in IHD patients using the Depression Assessment in Ischemic Heart Disease Questionnaire (DA-IHDQ) and to explore gender differences in depression severity and help-seeking behavior. **Methods**: This cross-sectional study involved 103 patients diagnosed with IHD (62 males, 41 females), with data collected from two general practice clinics in Arad, Romania, between November 2023 and November 2024. Participants completed the DA-IHDQ, a self-developed questionnaire designed to screen for depression in IHD patients. The questionnaire categorizes depression severity into four grades: minimal to no depression, mild depression, moderate depression, and severe depression. The study also assessed participants’ interest in receiving psychological support. Descriptive and inferential statistical analyses were performed, and the psychometric properties of DA-IHDQ, including its reliability (Cronbach’s α = 0.957) and diagnostic accuracy (sensitivity = 90.0%, specificity = 98.8%), were evaluated. **Results**: Mild depression was the most common grade in both male and female IHD patients, while severe depression was the least prevalent. Males had a higher overall frequency of depression, with more cases of mild depression, whereas females had a higher proportion of moderate and severe depression. Additionally, males demonstrated significantly lower interest in psychological or psychiatric help, while females showed greater willingness to seek mental health support. The DA-IHDQ exhibited strong internal consistency and high diagnostic accuracy in identifying depressive symptoms in IHD patients. The DA-IHDQ exhibited strong internal consistency and high diagnostic accuracy in identifying depressive symptoms in IHD patients. **Conclusions**: The findings highlight the high prevalence of depression in IHD patients and the gender disparities in mental health engagement, emphasizing the need for targeted psychological interventions. The DA-IHDQ demonstrated strong psychometric properties and could serve as an effective screening tool for depression in IHD care. The DA-IHDQ demonstrated strong psychometric properties and could serve as an effective screening tool for depression in IHD care. Future research should explore the barriers to help-seeking among male IHD patients and develop gender-sensitive strategies to improve access to mental health services.

## 1. Introduction

Ischemic heart disease (IHD) remains one of the leading causes of morbidity and mortality worldwide, significantly affecting both physical and psychological well-being [1,2]. Depression is highly prevalent among IHD patients and is associated with worse clinical outcomes, including increased cardiovascular events, poor treatment adherence, and reduced quality of life [3,4,5,6]. The bidirectional relationship between IHD and depression, in which depression worsens cardiovascular symptoms and IHD contributes to the development of depressive symptoms, has been well-documented [7,8]. This association complicates disease management and underscores the need for improved screening and treatment strategies [9,10].

Despite this well-established link, depression remains underdiagnosed and undertreated in IHD patients, particularly in men, who are often reluctant to seek psychological support due to stigma, cultural barriers, and limited awareness of mental health’s role in chronic disease management. Gender differences in help-seeking behavior are significant, with men less likely to engage with mental health services despite experiencing similar levels of depression as women [11,12,13,14]. Addressing these disparities is essential for improving the overall management of IHD and reducing its psychological burden [5,15,16].

Several standardized depression screening tools, including the Beck Depression Inventory-II (BDI-II) and the Patient Health Questionnaire-9 (PHQ-9), have been widely used in IHD patients. Some of these tools have also been translated into Romanian [17]. However, they were not specifically designed for IHD patients and may not adequately distinguish between depressive symptoms and somatic manifestations of cardiovascular disease, leading to potential misclassification. Many depressive symptoms, such as fatigue, sleep disturbances, and appetite changes, overlap with the physiological symptoms of IHD, increasing the risk of false positives or underdiagnosis. Furthermore, existing tools do not account for IHD-specific psychosocial stressors, such as anxiety about future health risks, the emotional impact of a cardiac diagnosis, and the role of social support in recovery.

To address these limitations, we developed the Depression Assessment in Ischemic Heart Disease Questionnaire (DA-IHDQ), a screening tool specifically designed for IHD patients. Unlike general depression instruments, the DA-IHDQ differentiates between IHD-related somatic symptoms and depressive symptoms, reducing the risk of misdiagnosis. Additionally, it incorporates IHD-specific psychosocial stressors, such as emotional distress following a cardiac diagnosis and anxiety about future health risks, which are often overlooked by standard depression scales. Furthermore, the DA-IHDQ accounts for gender-based differences in help-seeking behaviors, allowing for more targeted mental health interventions that address the unique challenges faced by both male and female IHD patients.

A preliminary pilot study demonstrated that the DA-IHDQ effectively captured the psychological burden of IHD while maintaining strong reliability and validity. This initial study focused on patients diagnosed with both IHD and depression [18]. To further validate its utility, this study evaluates the DA-IHDQ in a cohort of IHD patients without a formal depression diagnosis and compares its performance with the BDI-II to assess convergent validity.

The primary objectives of this study are to assess the prevalence and severity of depression in IHD patients and to investigate gender differences in depressive symptoms and help-seeking behaviors. By refining and validating the DA-IHDQ, we aim to improve depression screening in IHD patients, facilitating earlier psychological interventions that can enhance both mental health and cardiovascular outcomes [19,20].

## 2. Materials and Methods

### 2.1. Study Design and Setting

This cross-sectional study aimed to assess the emotional and psychological experiences of patients diagnosed with IHD, focusing on the prevalence of depression and interest in psychological or psychiatric help. Data collection was conducted at two primary care clinics in Romania: Dr. Tasedan Dana S.R.L. and Dr. Toth Praxis S.R.L.

Initially, recruitment began at Dr. Tasedan Dana S.R.L. in November 2023. However, due to a limited number of eligible participants, an additional recruitment site, Dr. Toth Praxis S.R.L., was introduced in June 2024 to ensure a sufficient sample size. The data collection period spanned from November 2023 to November 2024.

Both clinics provide primary care services, including the management of chronic cardiovascular conditions. This setting allowed for the identification of IHD patients in routine clinical practice, facilitating a real-world assessment of depression screening and mental health support needs.

### 2.2. Study Population

This study included a total of 103 patients diagnosed with IHD, recruited from two primary care clinics in Arad, Romania: Dr. Tasedan Dana S.R.L. and Dr. Toth Praxis S.R.L. The study population comprised 62 male and 41 female patients. To ensure a balanced representation of different living environments, participants were equally distributed based on their place of residence, with 52 patients from urban areas and 51 from rural areas

All patients included in the study had a confirmed diagnosis of IHD and were recruited during routine consultations at the two participating clinics. Eligible patients who met the inclusion criteria and provided informed consent were invited to complete the study questionnaire. The study aimed to capture a diverse sample of IHD patients to assess depression prevalence and gender-based differences in help-seeking behavior.

### 2.3. Participant Recruitment and Response Rate

**Figure 1:**
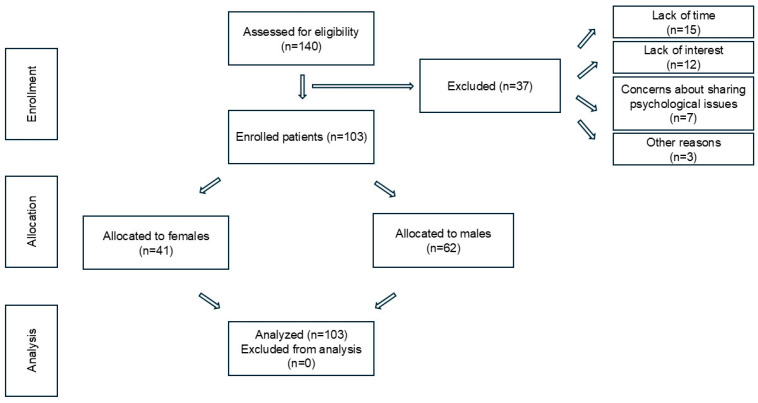
Recruitment flowchart of study participants.

A total of 140 IHD patients were invited to participate, with 103 (73.6%) completing the questionnaire (62 males, 41 females). The remaining 37 (26.4%) declined, primarily citing lack of time (40.5%), lack of interest (32.4%), and reluctance to discuss psychological symptoms (18.9%). The final sample ensured balanced urban and rural representation (Figure 1).

### 2.4. Inclusion and Exclusion Criteria

#### 2.4.1. Definitions

To ensure consistency in data collection and interpretation, the following definitions were applied in this study:

- Onset of IHD: Defined as the time elapsed since the patient’s first clinical diagnosis of IHD and confirmed by a physician based on clinical history, diagnostic tests (e.g., ECG, cardiac biomarkers, stress test), or imaging (e.g., coronary angiography, echocardiography). Patients were categorized into the following onset groups:
  ○ <1 month;
  ○ 1–3 months;
  ○ 3–6 months;
  ○ 6–12 months;
  ○ 1–3 years;
  ○ >3 years.
- Confirmed Diagnosis of IHD: Defined as a documented medical diagnosis of IHD, including chronic coronary syndrome (stable angina pectoris, silent myocardial infarction), acute coronary syndrome (unstable angina pectoris, acute myocardial infarction (AMI) with or without ST-segment elevation), or a history of myocardial revascularization (percutaneous coronary intervention or coronary artery bypass grafting). Diagnosis was based on clinical evaluation, imaging, and/or diagnostic tests.
- Genetic Factors: Defined as a family history of IHD or related cardiovascular conditions, including premature coronary artery disease, myocardial infarction, or stroke in first-degree relatives (parents or siblings). These hereditary predispositions are widely recognized as significant contributors to cardiovascular disease risk.

These definitions were used to ensure standardized classification across all participants before applying the inclusion and exclusion criteria.

#### 2.4.2. Inclusion Criteria

To ensure the relevance and representativeness of the study population, the following inclusion criteria were applied:

- Age: Participants were required to be adults aged 18 years or older.
- Gender: Both male and female patients were included in the study.
- Diagnosis of IHD: Patients with a confirmed diagnosis of IHD, based on the criteria detailed in Section 2.4.1.
- Willingness to Participate: Participants must be willing to take part in the study, with the understanding that they would be asked to complete questionnaires.
- Informed Consent: All patients must have provided written informed consent, indicating their understanding of the study’s objectives, procedures, potential risks, and the confidentiality of their data.

#### 2.4.3. Exclusion Criteria

To ensure the safety and accuracy of the findings, participants were excluded from the study based on the following criteria:

- Major Psychiatric Disorders: Patients with significant psychiatric disorders other than depression (e.g., schizophrenia, bipolar disorder) were excluded.
- No Evidence of IHD: Patients who did not meet the clinical, imaging, or diagnostic criteria for IHD were excluded.
- Cognitive Impairments or Language Barriers: Patients who were unable to comprehend the study questionnaires due to cognitive impairments (e.g., dementia or severe cognitive decline) or who faced language barriers.
- Pregnancy and Terminal Illness: Pregnant women and individuals in the terminal stages of any illness were excluded.
- Refusal to Participate or Lack of Consent: Any patient who refused to participate in the study or who did not provide informed consent was excluded.

By applying these inclusion and exclusion criteria, the study aimed to create a focused, homogeneous sample of IHD patients that would provide valid, reliable, and interpretable data regarding depression severity and the interest in psychological support among this patient group.

### 2.5. Data Collection Instruments

Data were collected using a structured questionnaire designed to assess the emotional and psychological impact of IHD. This questionnaire, known as the DA-IHDQ, was adapted from a pilot version and refined for broader application in this study. It aimed to evaluate depressive symptoms, emotional distress, coping mechanisms, and patients’ interest in psychological or psychiatric support. The questionnaire was structured into several sections, each addressing different aspects of the participants’ experience with IHD. The full DA-IHDQ is available in Supplementary Materials File S1, and a detailed psychometric validation report, including its reliability and validity analyses, is provided in Supplementary Materials File S2.

#### 2.5.1. Questionnaire Structure

The questionnaire was divided into several sections to comprehensively assess the emotional, psychological, and physical impacts of IHD on patients. Each section targeted specific aspects of the patient’s experience, from personal demographics to emotional responses and overall well-being, with a focus on both psychological distress and physical symptoms related to IHD.

1. Demographic Information: This section collected basic personal information, including gender, age, marital status, and social status.
2. Medical History of Heart Disease: This section assessed the type of IHD, its onset, and associated risk factors (e.g., hypertension, smoking, diabetes).
3. Emotional Responses to Diagnosis: Patients were asked about their feelings toward their diagnosis, changes in frustration, communication, and anxiety.
4. Daily Life and Functional Impact: This section assessed the impact of IHD on daily functioning, including work performance, physical symptoms, and ability to carry out daily tasks.
5. Future Outlook and Coping: Questions in this section focused on patients’ perspectives on their long-term health and quality of life.
6. General Well-Being: This section included questions on sleep quality, energy levels, appetite changes, health concerns, and thoughts of self-harm or suicide.

Following these sections, participants were asked about their interest in receiving psychological/psychiatric help.

The questionnaire was developed to address specific limitations of standard depression screening tools, particularly their inability to distinguish between depressive symptoms and cardiac-related somatic symptoms. By incorporating IHD-specific emotional distress and gender-based differences in help-seeking behaviors, the DA-IHDQ provides a targeted and clinically practical approach to depression screening in IHD patients.

#### 2.5.2. Depression Grade Interpretation

DA-IHDQ categorizes depression severity based on responses to specific items assessing emotional health, psychological distress, and somatic symptoms. The total score is derived from a 0–3 Likert scale, with higher scores indicating greater depressive symptom severity. Depression severity was classified into the following categories:

- 0–10: Minimal to no depression;
- 11–20: Mild depression;
- 21–30: Moderate depression;
- 31–42: Severe depression.

These cutoff points were established based on psychometric validation, ensuring that the DA-IHDQ accurately reflects clinically relevant levels of depression in IHD patients. The structured scoring system allows for the early detection of depressive symptoms, facilitating timely psychological interventions in routine cardiac care.

#### 2.5.3. Psychometric Validation

The DA-IHDQ was developed as a targeted screening tool for depressive symptoms in patients with IHD. To ensure its reliability and validity, the questionnaire underwent comprehensive psychometric evaluation, including tests for internal consistency, construct validity, criterion validity, and diagnostic accuracy.

The DA-IHDQ demonstrated strong psychometric properties, including high internal consistency (Cronbach’s α = 0.957) and robust construct validity. Exploratory factor analysis (EFA) with principal component analysis (PCA) and Varimax rotation confirmed a one-factor solution, which accounted for 61.8% of the total variance, indicating that the DA-IHDQ measures a unified construct of depression in IHD patients.

To establish criterion validity, the DA-IHDQ was compared with the BDI-II, a widely used and validated depression assessment tool. Pearson’s correlation analysis showed a strong positive correlation between the DA-IHDQ and BDI-II total scores (r = 0.935, *p* < 0.001), confirming that the DA-IHDQ reliably measures depressive symptoms in IHD patients.

Additionally, the DA-IHDQ exhibited high diagnostic accuracy, with an overall classification accuracy of 97.1%. Sensitivity (90.0%) and specificity (98.8%) values indicate that the DA-IHDQ is an effective screening tool for identifying depression in IHD patients.

Despite its strong psychometric performance, further validation in larger, multi-center studies is recommended to enhance its generalizability across diverse populations and clinical settings. The DA-IHDQ has the potential to be an essential tool for integrating mental health screening into routine cardiac care, promoting a comprehensive approach to patient well-being.

### 2.6. Ethical Considerations

This study was conducted in accordance with ethical guidelines to ensure the protection, confidentiality, and voluntary participation of all patients. Ethical approval was obtained from the Institutional Review Boards of the two primary care clinics: Dr. Tasedan Dana S.R.L. (Approval No. 48/07 November 2022) and Dr. Toth Praxis S.R.L. (Approval No. 14/24 June 2024). Additionally, ethical approval was granted by the Scientific Research Ethics Committee of the Faculty of Medicine and Pharmacy, University of Oradea (Approval No. CEFMF/3/30 September 2024).

Before participation, all patients received detailed information about the study’s objectives, procedures, and potential risks. Written informed consent was obtained from each participant, ensuring they fully understood their rights and the voluntary nature of their involvement.

To maintain confidentiality and anonymity, all collected data were coded and securely stored, ensuring that no personally identifiable information could be linked to individual responses. Participants were assured that their participation was entirely voluntary and they could withdraw from the study at any time without consequences.

Furthermore, special precautions were taken when addressing sensitive topics, such as depression severity and self-harm risk. Participants were provided with appropriate supportive resources and referrals if necessary to safeguard their well-being.

### 2.7. Study Objectives and Hypotheses

The following hypotheses were formulated to guide the investigation into the relationship between IHD and depression, as well as the potential gender-based differences in emotional and psychological responses:

1. Primary Hypothesis:

**H1.**  *Patients diagnosed with IHD will exhibit a higher prevalence of depression compared to the general population, with varying levels of depression severity (minimal, mild, moderate, severe) identified through the questionnaire. This hypothesis aims to test whether IHD contributes to the development or exacerbation of depressive symptoms, which is common in individuals dealing with chronic or serious medical conditions such as heart disease.*

2. Secondary Hypotheses:

**H2.**  *There will be a significant difference in the distribution of depression severity between male and female patients with IHD, with females showing higher levels of depression than males. This hypothesis explores the possibility of gender-based differences in the emotional responses to IHD, potentially influenced by both biological and socio-cultural factors.*

**H3.**  *Males with IHD will demonstrate a lower interest in receiving psychological or psychiatric help compared to females. This hypothesis is based on the observation that men may be less likely to seek help for mental health issues, especially those related to emotional well-being, which can influence their overall treatment and care experience.*

**H4.**  *There will be a positive correlation between the severity of depression and the lack of interest in receiving psychological or psychiatric help in both male and female patients with IHD. This hypothesis suggests that patients who exhibit more severe depression may be less likely to seek help, possibly due to stigma, lack of awareness, or inadequate access to mental health services.*

**H5.**  *Patients with IHD who experience more severe depression will report a greater functional impact of their heart condition on daily activities (e.g., work performance, physical activities, social interactions). This hypothesis aims to determine whether depression severity is associated with the degree to which IHD affects an individual’s daily life, including both physical and emotional aspects.*

**H6.**  *Gender differences will also be reflected in the functional impact of IHD on daily life, with women possibly reporting a higher impact on their physical and mental well-being compared to men. This hypothesis tests whether gender influences how IHD affects daily life, potentially due to different social roles, coping mechanisms, and health behaviors in men and women.*

These hypotheses will be tested through the data gathered from patient questionnaires, focusing on depression severity, gender differences, interest in psychological support, and the relationship between emotional health and the functional impact of IHD.

### 2.8. Data Analysis

Statistical analyses were conducted using JASP 0.19.1 (University of Amsterdam, Amsterdam, The Netherlands) for general statistical tests and Jamovi 2.3.28 (The Jamovi Project, Sydney, NSW, Australia) for diagnostic accuracy analysis.

The following statistical methods were applied:

- Descriptive statistics (means, standard deviations, frequency distributions);
- Independent *t*-tests and chi-square tests to analyze gender differences in depression severity and help-seeking behavior;
- Spearman’s rank correlation to assess relationships between depression severity and interest in psychological/psychiatric support;
- Cronbach’s alpha to assess internal consistency (≥0.9 indicating excellent reliability);
- EFA with PCA and Varimax rotation to assess construct validity;
- Parallel analysis to confirm factor retention;
- Pearson’s correlation between DA-IHDQ and BDI-II to assess criterion validity;
- Confusion matrix interpretation, positive predictive value (PPV), negative predictive value (NPV), and likelihood ratios (LR+ and LR−) to determine diagnostic accuracy.

The statistical significance threshold was set at *p* < 0.05. All statistical tests related to the validation of the DA-IHDQ have been included in Supplementary Materials File S2.

## 3. Results

This section presents a comprehensive analysis of the impact of IHD on patients’ general well-being, emotional health, and attitudes toward psychological support. It explores gender-specific differences in sleep quality, energy levels, appetite changes, health-related concerns, and thoughts of self-harm or suicide. Additionally, the findings highlight correlations among these variables, emphasizing their interrelated nature. Depression grades and interest in psychological or psychiatric help are compared between male and female patients, offering insights into their mental health needs and preferences for support. These results provide valuable context for understanding the broader psychosocial impact of IHD.

### 3.1. The Impact of Demographic Information on the IHD–Depression Relationship

The study included 103 patients diagnosed with IHD, with a gender distribution of 41 females (39.8%) and 62 males (60.2%) (Table 1). A significant association was observed between gender and age (*p* < 0.001), with females predominantly in the 60–79 age group and males in the 40–59 age group. Marital status also differed by gender (*p* = 0.006), as females were more likely to be widowed or divorced, whereas males were more frequently married. Additionally, a significant relationship was found between gender and social status (*p* = 0.011), with females more likely to be retired or on a disability pension, while males were more commonly employed (Table 2).

### 3.2. Medical History of Heart Disease

A significant association was found between gender and the type of IHD (*p* < 0.001). AMI was more prevalent in males (62.9%) compared to females (29.3%), while stable angina pectoris (SAP) and silent myocardial infarction (SMI) were more frequent in females (39.0% and 19.5%, respectively). Unstable angina pectoris (UAP) was also more common in males (25.8%) than in females (12.2%) (Table 3). The strength of this association was confirmed by a Contingency Coefficient of 0.457 and Cramér’s V of 0.513, suggesting a strong relationship between gender and IHD type (Table 4).

### 3.3. Gender Differences in Medical History and Risk Factors in IHD Patients

Significant gender differences were observed in several medical risk factors associated with IHD (Table 5). Hypertension was highly prevalent in both genders, with a significant association (*p* < 0.001 for females, *p* = 0.003 for males). Smoking and alcohol abuse were significantly more common among males, with 87.1% of men smoking compared to 31.7% of women (*p* < 0.001 and *p* = 0.028, respectively). Similarly, alcohol consumption was reported in 56.5% of males compared to 22% of females (*p* < 0.001).

Obesity was significantly more prevalent in females (68.3%) than males (53.5%), with a significant association in females (*p* = 0.028). Hypercholesterolemia was a major risk factor for both genders, present in 75.6% of females and 74.2% of males, with strong statistical significance (*p* = 0.001 for females, *p* < 0.001 for males). Hypertriglyceridemia was significantly associated with IHD in males (*p* = 0.007). Inflammation was also a notable factor, affecting 70.7% of females and 66.1% of males, with strong statistical significance (*p* = 0.012 for females, *p* = 0.015 for males).

Additionally, genetic predisposition was significantly associated with IHD in females (*p* = 0.012), with 70.7% of women reporting a family history of cardiovascular disease. These findings suggest that while traditional cardiovascular risk factors are present in both genders, certain factors such as smoking, alcohol abuse, and hypertriglyceridemia are more prevalent in males, whereas obesity and genetic predisposition appear to have a stronger impact in females (Table 5).

### 3.4. Emotional Reactions to Diagnosis by Gender

Significant gender differences were observed in emotional responses to an IHD diagnosis (Table 6). Sadness related to the diagnosis was commonly reported in both genders, with 39.0% of females and 32.3% of males experiencing occasional overwhelming sadness. However, a higher proportion of males (21.0%) reported deep sadness they could not overcome compared to females (14.6%).

Frustration and anger increased in both groups, with 31.7% of females and 30.6% of males reporting that small things triggered their anger. However, constant anger was slightly more frequent in males (12.9%) than in females (9.8%).

Social withdrawal and communication difficulties were more pronounced in females, as 39.0% of women reported feeling detached and indifferent to social interactions compared to 24.2% of males. Males, however, were slightly more likely to report mild difficulty expressing their feelings (50.0% vs. 41.5% in females).

Anxiety related to the diagnosis was high in both groups, with 39.0% of females and 38.7% of males experiencing frequent anxiety. Additionally, 9.8% of females and 8.1% of males reported constant overwhelming anxiety. These findings suggest that while both genders experience high levels of distress, males may be more prone to deep sadness, while females are more likely to withdraw socially (Table 6).

Significant positive correlations were observed among all emotional response variables, indicating that sadness, frustration, communication difficulties, and anxiety are closely interconnected in IHD patients (Table 7).

Sadness about diagnosis (Q1) correlated strongly with frustration (Q2) (ρ = 0.591, *p* < 0.001), communication difficulties (Q3) (ρ = 0.654, *p* < 0.001), and anxiety (Q4) (ρ = 0.542, *p* < 0.001). This suggests that patients who experience greater sadness also report higher frustration, difficulty expressing emotions, and elevated anxiety.

Frustration (Q2) was significantly associated with both communication difficulties (Q3) (ρ = 0.517, *p* < 0.001) and anxiety (Q4) (ρ = 0.562, *p* < 0.001), indicating that heightened frustration is linked to increased emotional distress and social withdrawal.

Communication difficulties (Q3) had a strong positive correlation with anxiety (Q4) (ρ = 0.648, *p* < 0.001), reinforcing that patients who struggle to express their emotions also experience higher anxiety levels.

### 3.5. Gender Differences in the Impact of Heart Disease on Work and Daily Functioning

Females reported greater challenges in maintaining work performance and motivation following an IHD diagnosis compared to males. Only 9.8% of females maintained their pre-diagnosis work performance, whereas 27.4% of males reported no changes. The majority of participants indicated needing extra effort to fulfill work responsibilities (46.3% of females, 38.7% of males), while more females (31.7%) struggled with motivation compared to males (22.6%).

Regarding physical symptoms, 56.1% of females and 45.2% of males reported minor discomfort but were able to manage daily activities. More females (31.7%) than males (29.0%) experienced frequent interference of physical symptoms in their daily tasks, whereas a higher proportion of males (11.3%) reported severe limitations compared to females (2.4%). These findings suggest that while both genders face functional challenges due to IHD, the impact on work motivation is more pronounced in females, whereas severe physical limitations are more common among males (Table 8).

There was a significant positive correlation between difficulties in work performance and motivation (Q5) and the impact of physical symptoms on daily activities (Q6), with a Spearman’s rho of 0.547 (*p* < 0.001). This suggests that individuals who experience greater challenges in maintaining work responsibilities are also more likely to report increased interference from physical symptoms in their daily activities. The moderate strength of this correlation highlights the interconnected nature of emotional and physical burdens in IHD patients, emphasizing the need for comprehensive care approaches that address both aspects (Table 9).

### 3.6. Gender Differences in the Impact of Heart Disease on Future Outlook, Quality of Life, and Romantic Relationships

Most patients reported occasional worry about their future health, with 61.0% of females and 53.2% of males expressing concerns, while a smaller proportion had low expectations or a bleak outlook regarding their long-term health (Table 10). Quality of life dissatisfaction was common, affecting 43.9% of females and 53.2% of males, though extreme dissatisfaction was less frequent. Regarding romantic relationships, 39.0% of females and 51.6% of males experienced a slight decrease in interest, while a smaller percentage (26.8% of females, 22.6% of males) reported a significant loss of interest in intimacy. These findings suggest that while IHD impacts future outlook, quality of life, and romantic relationships, the severity varies among individuals.

A significant positive correlation was observed between future outlook and quality of life (ρ = 0.539, *p* < 0.001), suggesting that patients with a more optimistic perspective on their health tend to report higher life satisfaction. Additionally, future outlook was strongly correlated with interest in romantic relationships or intimacy (ρ = 0.702, *p* < 0.001), indicating that individuals with a positive perception of their future are more likely to maintain romantic connections. A moderate to strong correlation was also found between quality of life and romantic interest (ρ = 0.593, *p* < 0.001), highlighting the relationship between overall well-being and social or emotional engagement (Table 11).

### 3.7. Gender Differences in the Impact of Heart Disease on General Well-Being

Sleep disturbances were reported by the majority of participants, with males more frequently experiencing difficulty falling asleep and staying asleep (50.0%) compared to females (34.1%). Fatigue was a common concern, with 46.3% of females and 46.8% of males feeling tired more quickly, while 9.8% of females and 16.1% of males reported extreme exhaustion. Appetite loss was more frequent in females (34.1%) than in males (19.4%). Concerns about overall health were prevalent, with 36.6% of females and 24.2% of males stating that health issues often dominated their thoughts. Although the majority of participants denied thoughts of self-harm, 24.4% of females and 14.5% of males reported occasional thoughts, and a concerning minority (9.8% of females and 8.1% of males) admitted to serious suicidal ideation (Table 12).

Significant correlations were observed among all general well-being variables, indicating their interrelated nature in IHD patients. Poor sleep quality was strongly associated with increased fatigue (ρ = 0.613, *p* < 0.001), appetite changes (ρ = 0.690, *p* < 0.001), heightened health concerns (ρ = 0.736, *p* < 0.001), and greater emotional distress, including suicidal thoughts (ρ = 0.802, *p* < 0.001). Fatigue correlated positively with appetite loss (ρ = 0.643, *p* < 0.001) and emotional distress (ρ = 0.714, *p* < 0.001). Appetite changes were strongly associated with heightened health concerns (ρ = 0.715, *p* < 0.001) and thoughts of self-harm (ρ = 0.740, *p* < 0.001). The strongest correlation was observed between concerns about overall health and suicidal ideation (ρ = 0.815, *p* < 0.001), emphasizing the substantial psychological burden experienced by IHD patients (Table 13).

### 3.8. Reliability Analysis

The reliability analysis demonstrated excellent internal consistency for the DA-IHDQ, with a Cronbach’s alpha of 0.957 (95% CI: 0.943–0.968), confirming that the questionnaire items consistently measure the same construct (Table 14). The individual item reliability analysis indicated that removing any single item would result in only a minimal change in Cronbach’s alpha (ranging from 0.952 to 0.955), suggesting that all items contribute meaningfully to the overall reliability of the scale (Table 15).

### 3.9. Construct Validity

The chi-square goodness-of-fit test indicated an acceptable model fit (χ2 = 98.069, p = 0.053) (Table 16). Factor loadings ranged from 0.706 to 0.876, with the highest loadings observed for items related to health concerns and emotional distress (Table 17), reinforcing the questionnaire’s focus on psychological responses to IHD. EFA confirmed a one-factor structure for the DA-IHDQ, accounting for 61.8% of the total variance (Table 18). Parallel analysis further supported the one-factor model, as the first factor’s eigenvalue (9.021) exceeded the simulated threshold (1.694), while subsequent factors had eigenvalues below the corresponding simulated values, confirming the retention of a single latent construct (Table 19).

### 3.10. Criterion Validity

A strong positive correlation was found between DA-IHDQ and BDI-II scores (r = 0.935, *p* < 0.001), confirming the criterion validity of the DA-IHDQ as a reliable tool for assessing depressive symptoms in IHD patients (Table 20). This result demonstrates that DA-IHDQ scores align closely with the well-established BDI-II, supporting its effectiveness as a screening instrument for depression in this population.

### 3.11. Sensitivity and Specificity

The diagnostic performance of the DA-IHDQ was evaluated using a confusion matrix, which summarizes the classification outcomes (Table 21). The sample distribution further details the classification statistics (Table 22). The DA-IHDQ demonstrated high diagnostic accuracy, correctly classifying 97.1% of cases. Sensitivity was 90.0%, indicating a strong ability to detect individuals with depression, while specificity was 98.8%, effectively minimizing false positives. The PPV was 94.7%, and the NPV was 97.6%, confirming the test’s reliability in distinguishing depressed and non-depressed individuals. The LR+ (74.7) suggests that a positive test result strongly indicates depression, whereas the LR− (0.101) effectively rules it out (Table 23).

### 3.12. Distribution of Depression Grades in Males and Females Diagnosed with IHD

**Figure 2:**
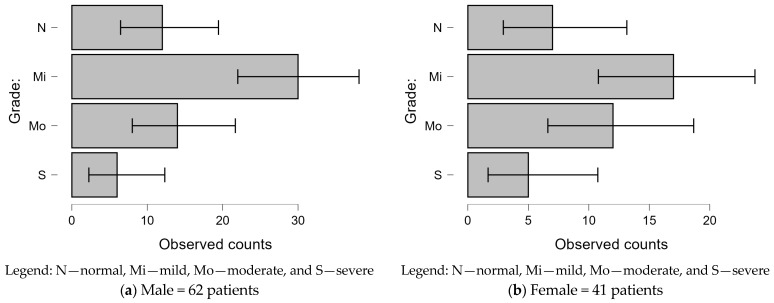
Gender-based comparison of depression grades in patients with IHD.

Mild depression was the most common grade in both male and female IHD patients. Males had a higher proportion of mild depression (48.4%) compared to females (41.4%), while females exhibited a higher prevalence of moderate depression (29.3%) compared to males (22.6%). Severe depression was observed in a small percentage of both groups, with a slightly higher proportion in females (12.2%) than males (9.7%). These findings highlight gender differences in depression severity among IHD patients (Figure 2).

### 3.13. Gender Differences in Interest in Psychological/Psychiatric Help Among Patients with IHD

**Figure 3:**
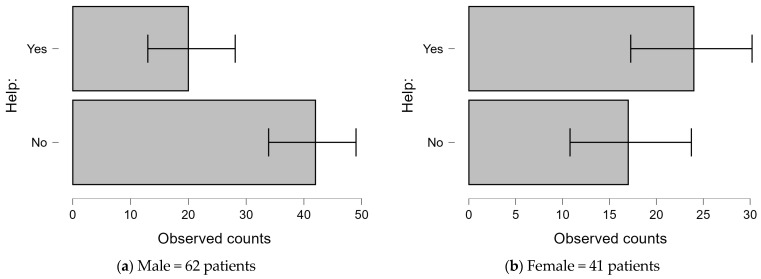
Interest in receiving psychological/psychiatric help among males and females diagnosed with IHD.

A significant gender difference was observed in the willingness to seek psychological or psychiatric help (Figure 3). Among males, 67.7% (42 individuals) declined psychological support, while only 32.3% (20 individuals) expressed interest. In contrast, 58.5% (24 individuals) of females were willing to receive psychological or psychiatric help, with 41.5% (17 individuals) reporting no interest. These findings indicate that female patients are more likely than males to seek mental health support in the context of IHD.

## 4. Discussion

This study aimed to investigate the prevalence and severity of depression among patients diagnosed with IHD and explore the gender differences in emotional responses to the condition, as well as the level of interest in psychological or psychiatric help. The hypotheses formulated at the onset of the study were generally supported by the observed results, with several key findings that contribute to understanding the intersection between IHD and mental health.

The high reliability of DA-IHDQ underscores its value as a clinical screening tool. These findings emphasize the need for integrating mental health services into cardiac care pathways, particularly for patients with depressive symptoms. Additionally, the study highlights the importance of gender-sensitive approaches in care delivery, addressing both the emotional and physical aspects of depression.

### 4.1. Prevalence of Depression in IHD Patients

As hypothesized, the results confirmed that patients diagnosed with IHD exhibit a higher prevalence of depression compared to the general population. The majority of participants in both male and female groups were found to experience mild depression, followed by moderate and minimal depression, with severe depression being the least common. This finding aligns with previous studies showing a high rate of depressive symptoms in individuals with cardiovascular diseases, including IHD [21,22,23]. For example, research has shown that depression is prevalent among patients with coronary artery disease and is associated with poorer health outcomes, including a heightened risk of mortality [24,25]. The relationship between chronic illness and depression is complex and can be influenced by multiple factors, such as the stress of living with a chronic condition, social isolation, and changes in daily life and physical functioning [26,27,28].

Moreover, the observed severity distribution of depression, where mild depression was most prevalent, underscores the importance of screening for mental health symptoms in IHD patients. These results suggest that while severe depression may not be as common, even mild to moderate depression can significantly impact the well-being of patients and may require attention from healthcare providers.

### 4.2. Gender Differences in Depression Severity

The study’s hypothesis regarding gender-based differences in depression severity was supported by the findings. Both male and female IHD patients most commonly experienced mild depression, with severe depression being the least prevalent. Males showed a higher overall frequency of depression, particularly in the mild depression category, while females had a higher proportion of moderate depression. Although both genders exhibited low rates of severe depression, females had a slightly higher proportion in this category. This result is consistent with the broader body of literature that suggests women may be more susceptible to depression than men, particularly in the context of chronic illness [29,30,31]. Previous research has shown that females with cardiovascular conditions tend to experience worse mental health outcomes compared to their male counterparts, which may be attributed to hormonal differences, social and psychological factors, and gendered expectations related to caregiving roles [32,33,34].

The observed higher counts of depression in females could also reflect gender-specific coping mechanisms or a greater willingness among women to report emotional distress. In contrast, males may underreport depression or engage in more socially acceptable expressions of distress, which can mask the true extent of their emotional suffering [35,36]. Future studies may benefit from further exploring how gender-specific factors influence both the reporting of depression and the actual experience of emotional distress in IHD patients.

### 4.3. Severity Interest in Psychological or Psychiatric Help

Our hypothesis that males with IHD would demonstrate a lower interest in seeking psychological or psychiatric help was supported by the findings. Males were less likely to express interest in mental health services, with a clear majority indicating no desire to seek psychological assistance. This finding is in line with existing research on gender differences in seeking mental health support. Men, particularly those with chronic illnesses such as heart disease, are often less likely to seek mental health services [37,38]. This reluctance is frequently attributed to the stigma surrounding mental health and societal expectations that men should remain stoic and self-reliant [39,40]. This underscores the need for targeted interventions that address these gender-based barriers to mental health care, including education and support strategies that reduce stigma and encourage help-seeking behaviors among men [41,42,43].

Conversely, females with IHD demonstrated a higher level of interest in receiving psychological or psychiatric help, supporting our hypothesis that women are more likely to seek mental health services. This trend has been consistently observed in the literature, with women generally being more open to discussing mental health issues and seeking help for emotional problems [44,45]. Despite this, the variability in both genders’ responses suggests that tailored interventions are necessary to address the individual needs of patients, particularly with regard to mental health support.

### 4.4. Impact of Depression Severity on Functional Outcomes

The relationship between depression severity and the functional impact of IHD on daily activities, as hypothesized, was evident in the results. Patients with higher depression scores reported greater difficulties in performing daily tasks, particularly in work performance, physical activities, and social interactions. This finding reinforces the idea that depression exacerbates the functional burden of chronic conditions like IHD. Patients with moderate to severe depression reported significantly higher levels of impairment in their ability to manage daily activities, which aligns with research indicating that depression can impair cognitive function, energy levels, and overall motivation [46,47,48].

The finding that depression severity correlates with functional limitations highlights the need for integrated care models that address both the physical and emotional aspects of chronic disease. Healthcare providers should recognize the importance of mental health care in improving the overall quality of life and functional outcomes for IHD patients. Screening for depression should, therefore, be a routine part of IHD management, and patients with moderate to severe depression should be referred for appropriate psychological or psychiatric treatment.

### 4.5. Future Research Directions

This study provides important insights into the psychological burden of IHD, highlighting the need for further research on the long-term impact of depression on cardiac outcomes and the development of effective, gender-sensitive interventions. Given the strong association between depression and adverse cardiovascular events, future studies should focus on longitudinal analyses to determine whether depression exacerbates IHD progression, influences treatment adherence, or increases mortality risk. Prospective cohort studies tracking depressive symptoms over time could provide causal evidence for this relationship and guide early intervention strategies.

One critical area for future investigation is the development and assessment of tailored mental health interventions for IHD patients, particularly for men, who demonstrate lower help-seeking behavior. Research should explore strategies to improve engagement with psychological care, including peer-support programs, digital mental health tools, and integrated psychosocial interventions within cardiac rehabilitation programs. Additionally, given the observed gender differences in emotional distress and social isolation, future research should refine gender-specific therapeutic approaches that address the distinct psychological needs of male and female patients.

Further studies should also examine cultural and socioeconomic factors that shape attitudes toward mental health support in IHD patients. Differences in healthcare access, stigma, and social determinants of health may significantly influence the diagnosis and management of depression in this population. Expanding research across diverse geographic regions and healthcare systems will improve the generalizability of findings and inform more inclusive, patient-centered care models.

Another crucial direction for future research involves expanding the scope of mental health assessments in IHD patients. Given the frequent co-occurrence of anxiety and depression, future iterations of the DA-IHDQ could include an anxiety screening component to evaluate the interplay between these conditions and their combined impact on cardiovascular outcomes. Additionally, studies comparing the DA-IHDQ with other validated depression scales in different clinical settings will further establish its diagnostic accuracy and clinical utility.

Finally, future research should focus on external validation and refinement of the DA-IHDQ in larger, multi-center studies. While this study demonstrates strong psychometric properties, further testing in diverse patient populations will confirm its robustness as a screening tool. Comparative studies evaluating its performance against gold-standard depression assessments will provide further validation and optimization of its use in routine clinical practice. By refining mental health screening and intervention strategies, future research can contribute to more comprehensive, multidisciplinary approaches for the management of IHD, ultimately improving both psychological well-being and cardiovascular outcomes in this high-risk population.

### 4.6. Implications for Clinical Practice

This study underscores the urgent need to integrate mental health care into IHD management, particularly through gender-sensitive interventions that address the distinct psychological and social challenges faced by male and female patients. Given the high prevalence of depression in IHD patients and its impact on treatment adherence, cardiac outcomes, and quality of life, a multidisciplinary approach combining cardiology, psychiatry, and psychology is essential.

1. Routine depression screening should be incorporated into cardiology, primary care, and cardiac rehabilitation programs to ensure early detection and timely intervention. The DA-IHDQ, with its high sensitivity (90.0%) and specificity (98.8%), can serve as a reliable screening tool in these settings. In cardiology clinics, screening during routine check-ups can help monitor emotional well-being. In primary care, early identification of depressive symptoms can facilitate timely referrals to mental health specialists. In cardiac rehabilitation, integrating structured psychological screening ensures a comprehensive evaluation of both physical and emotional health.
2. Gender-sensitive mental health interventions should address differences in depression severity and help-seeking behaviors. Male patients demonstrate low engagement in mental health care, necessitating proactive screening and psychoeducation about the link between depression and cardiovascular health. Peer support programs and structured cognitive–behavioral therapy (CBT) could improve engagement. Female patients experience higher emotional distress, social isolation, and caregiving responsibilities, highlighting the need for integrated psychological services within cardiac rehabilitation programs that focus on stress reduction, social support, and psychotherapy.
3. A multidisciplinary care model involving cardiologists, psychiatrists, psychologists, and social workers can optimize patient-centered care. Routine psychological assessments in cardiology consultations can improve early identification of depression. Coordinated treatment plans integrating antidepressant therapy, CBT, and lifestyle modifications can enhance both mental health and cardiac outcomes. Patient education programs should emphasize the bidirectional relationship between depression and IHD, improving awareness and treatment adherence.
4. Integrating mental health into cardiac rehabilitation can enhance patient outcomes by incorporating structured psychological interventions. CBT-based behavioral interventions can help patients manage stress, anxiety, and depression. Mindfulness-based stress reduction (MBSR) techniques can improve emotional resilience. Personalized rehabilitation plans should consider gender-specific psychological responses and adapt exercise, nutrition, and counseling strategies accordingly.
5. Training healthcare professionals in cardio-psychological care is essential for equipping them with the skills to recognize and manage depression in cardiac patients. Clinicians should be trained in screening for depression in cardiology settings, utilizing gender-sensitive communication to encourage psychological support, and implementing integrated care models that bridge mental health and cardiovascular management.

The findings of this study reinforce the need for a holistic, gender-sensitive approach to IHD management, integrating routine depression screening, tailored psychological interventions, and multidisciplinary collaboration. By embedding mental health support into cardiac care, healthcare providers can enhance treatment adherence, improve quality of life, and promote better long-term cardiovascular outcomes. Future research should focus on refining personalized treatment strategies and expanding multi-center validation studies to ensure the broader applicability of these interventions.

### 4.7. Research Limitations

While this study provides valuable insights into the relationship between depression and IHD, several limitations must be acknowledged, as they may influence the interpretation of findings and highlight areas for future research to refine and validate the DA-IHDQ.

- Cross-sectional design: This study captures data at a single point in time, limiting the ability to establish causal relationships between depression and IHD. It remains unclear whether depression contributes to the progression of IHD or whether IHD exacerbates depressive symptoms. A longitudinal study would be necessary to track depression severity over time and assess its long-term impact on cardiovascular outcomes.
- Self-reported data and potential bias: The study relies on patient-reported measures, which are susceptible to recall bias and social desirability bias. Men, in particular, may underreport depressive symptoms due to stigma, potentially affecting the accuracy of observed gender differences. Future studies should incorporate clinician-administered psychiatric evaluations, structured interviews, or biomarkers of stress and depression to enhance data accuracy.
- Limited sample size and generalizability: The sample size, though adequate for preliminary analysis, was restricted to patients from two clinical sites in Arad, Romania, potentially limiting the generalizability of findings. The homogeneity of the study population may not reflect broader demographic, socioeconomic, and cultural variations in depression among IHD patients. Future research should include larger, multi-center cohorts from diverse healthcare settings to improve external validity.
- Exclusion of patients with severe psychiatric disorders: To focus specifically on depression within the context of IHD, patients with severe psychiatric comorbidities (e.g., schizophrenia, bipolar disorder) were excluded. This may have led to an underestimation of depression prevalence in cardiac patients. Future research should assess how severe psychiatric conditions interact with IHD-related depression while implementing appropriate methodological controls.
- Cultural and gender considerations in mental health reporting: Cultural attitudes toward mental health and gender norms may have influenced how participants reported depressive symptoms and help-seeking behaviors. In many settings, men are less likely to express emotional distress, which could lead to an underestimation of depression severity. Future studies should explore how cultural factors influence depression severity, coping mechanisms, and mental health service utilization across diverse population
- Challenges in clinical implementation: While the DA-IHDQ has demonstrated strong psychometric properties, its practical integration into routine cardiac care may face logistical challenges, such as time constraints in cardiology consultations and the need for specialized training among healthcare providers. Future research should evaluate the feasibility of incorporating DA-IHDQ into real-world clinical workflows and identify potential barriers to its widespread adoption.

Despite these limitations, this study contributes meaningful insights into the psychological burden of IHD and underscores the necessity of integrating mental health care into routine cardiac management. The DA-IHDQ offers a condition-specific approach that differentiates between cardiac-related somatic symptoms and psychological distress, enhancing the accuracy of depression screening.

Future research should focus on validating the DA-IHDQ in larger, multi-center studies and evaluating its effectiveness in routine clinical practice, particularly in high-risk IHD populations. Additionally, further investigation into gender-sensitive interventions and culturally adapted screening approaches can refine mental health strategies for IHD patients. The strong psychometric properties of the DA-IHDQ highlight its potential as a valuable tool for routine depression screening in cardiology settings.

## 5. Conclusions

This study emphasizes the significant prevalence of depression among patients with IHD, revealing notable gender-based differences in symptom presentation and psychosocial impact. DA-IHDQ was found to be a reliable and effective tool for screening depression, offering a condition-specific approach that distinguishes cardiac-related somatic symptoms from psychological distress. Its high sensitivity and specificity underscore its potential for routine implementation in cardiology settings, facilitating early detection and intervention.

The study supports the hypothesis that males with IHD exhibit lower interest in seeking psychological or psychiatric help compared to females, emphasizing the need for targeted mental health interventions that address gender-specific barriers. Furthermore, the functional limitations experienced by patients with higher depression severity underscore the importance of integrated care models that address both the physical and emotional aspects of chronic illness.

Given the impact of depression on IHD outcomes, routine screening should become a standard component of cardiac care, with healthcare providers trained to recognize and manage depression effectively. Multidisciplinary collaboration among cardiologists, psychiatrists, and psychologists is essential for optimizing treatment adherence and improving patient well-being. Expanding mental health components within cardiac rehabilitation programs, particularly through CBT-based interventions, psychoeducation, and gender-sensitive approaches, can significantly enhance patient outcomes.

Future research should focus on validating the DA-IHDQ in larger, multi-center cohorts, assessing its effectiveness in longitudinal monitoring, and exploring integrated interventions that combine pharmacological, psychological, and lifestyle-based approaches. Additionally, further investigation into the co-occurrence of anxiety with depression in IHD patients will provide a more comprehensive understanding of their mental health needs.

By recognizing and addressing the mental health challenges faced by individuals with IHD, healthcare providers can enhance patient care, improve cardiovascular outcomes, and ultimately reduce the burden of both cardiac and mental health comorbidities.

## Figures and Tables

**Table 1:** Demographic characteristics of study participants.

		Gender = 103 Patients	
Variable	Level	Female = 41 Patients (39.8%)	Male = 62 Patients (60.2%)
Age	<40	0 (0.00%)	6 (9.7%)
	40–59	10 (24.4%)	31 (50.0%)
	60–79	28 (68.3%)	25 (40.3%)
	80–89	3 (7.3%)	0 (0.00%)
Environment	Urban	21 (51.2%)	31 (50.0%)
	Rural	20 (48.8%)	31 (50.0%)
Marital status	Married	17 (41.5%)	43 (69.4%)
	Single	3 (7.3%)	4 (6.5%)
	Widowed	14 (34.1%)	10 (16.1%)
	Divorced	7 (17.1%)	2 (3.2%)
Social status	Employed	8 (19.5%)	22 (35.5%)
	Disability Pension	16 (39.0%)	14 (22.6%)
	Unemployed	1 (2.4%)	11 (17.7%)
	Age Pension	16 (39.0%)	15 (24.2%)

**Table 2:** Associations between demographic variables and gender.

	Chi-Squared Test			Contingency Coefficient	Cramer’s V
Variable	Value	df	*p*		
Age	16.323	3	<0.001	0.370	0.398
Environment	0.015	1	0.904	0.012	0.012
Marital status	12.551	3	0.006	0.330	0.349
Social status	11.217	3	0.011	0.313	0.330

**Table 3:** Distribution of IHD types and onset duration by gender.

		Gender = 103 Patients	
Variable	Level	Female = 41 Patients (39.8%)	Male = 62 Patients (60.2%)
Type of Ischemic Heart Disease	Unstable Angina Pectoris	5 (12.2%)	16 (25.8%)
	Acute Myocardial Infarction	12 (29.3%)	39 (62.9%)
	Stable Angina Pectoris	16 (39.0%)	3 (4.8%)
	Silent Myocardial Infarction	8 (19.5%)	4 (6.5%)
Onset of Ischemic Heart Disease	<1 month	7 (17.1%)	14 (22.6%)
	1–3 months	10 (24.4%)	12 (19.4%)
	3–6 months	6 (14.6%)	12 (19.4%)
	6–12 months	6 (14.6%)	8 (12.9%)
	1–3 year	8 (19.5%)	10 (16.1%)
	>3 year	4 (9.8%)	6 (9.7%)

**Table 4:** Chi-square test results for gender and IHD Characteristics.

	Chi-Squared Test			Contingency Coefficient	Cramer’s V
Variable	Value	df	*p*		
Type of Ischemic Heart Disease	27.130	3	<0.001	0.457	0.513
Onset of Ischemic Heart Disease	1.191	5	0.946	0.107	0.108

**Table 5:** Prevalence of medical conditions and risk factors by gender in IHD patients.

		Female = 41 Patients		Male = 62 Patients	
Variable	Level	Counts	*p*	Counts	*p*
Hypertension	No	9 (22%)	<0.001	19 (30.6%)	0.003
	Yes	32 (78%)	<0.001	43 (69.4%)	0.003
Smoking	No	28 (68.3%)	0.028	8 (12.9%)	<0.001
	Yes	13 (31.7%)	0.028	54 (87.1%)	<0.001
Alcohol	No	32 (78%)	<0.001	27 (43.5%)	0.107
	Yes	9 (22%)	<0.001	35 (56.5%)	0.107
Obesity	No	13 (31.7%)	0.028	46 (46.5%)	0.374
	Yes	28 (68.3%)	0.028	53 (53.5%)	0.374
Diabetes	No	15 (36.6%)	0.117	28 (45.2%)	0.526
	Yes	26 (63.4%)	0.117	34 (54.8%)	0.526
Hypercholesterolemia	No	10 (24.4%)	0.001	16 (25.8%)	<0.001
	Yes	31 (75.6%)	0.001	46 (74.2%)	<0.001
Hypertriglyceridemia	No	14 (34.1%)	0.060	20 (32.3%)	0.007
	Yes	27 (65.9%)	0.060	42 (67.7%)	0.007
Inflammation	No	12 (29.3%)	0.012	21 (33.9%)	0.015
	Yes	29 (70.7%)	0.012	42 (66.1%)	0.015
Tachycardia	No	18 (39.3%)	0.533	28 (45.2%)	0.526
	Yes	23 (56.1%)	0.533	34 (54.8%)	0.526
Genetic factors	No	12 (29.3%)	0.012	24 (38.7%)	0.098
	Yes	29 (70.7%)	0.012	38 (61.3%)	0.098

**Table 6:** Emotional responses to heart disease diagnosis by gender.

		Female 41 Patients	Male 62 Patients
Subsection 3: Emotional Responses to Diagnosis			
Variable	Level	Counts	Counts
Q1. Feelings about Heart Disease Diagnosis	0. I feel positive and confident about managing my condition.	5 (12.2%)	6 (9.7%)
	1. Sometimes I feel a bit sad about my diagnosis.	14 (34.1%)	23 (37.1%)
	2. Occasionally, I feel overwhelmed by sadness, but not all the time.	16 (39.0%)	20 (32.3%)
	3. I often feel a deep sadness that I can’t seem to overcome.	6 (14.6%)	13 (21.0%)
Q2. Changes in Frustration or Anger Since Diagnosis	0. I manage my emotions as I did before the diagnosis.	9 (22.0%)	16 (25.8%)
	1. I get frustrated more easily than before but rarely feel angry.	15 (36.6%)	19 (30.6%)
	2. I get irritated easily, and even small things can trigger my anger.	13 (31.7%)	19 (30.6%)
	3. I feel constantly angry because of my health issues.	4 (9.8%)	8 (12.9%)
Q3. Changes in Communication Since Diagnosis	0. I communicate as well as I did before my diagnosis.	6 (14.6%)	12 (19.4%)
	1. I find it slightly harder to express my feelings but still try to communicate.	17 (41.5%)	31 (50.0%)
	2. I feel indifferent to social interactions and somewhat detached.	16 (39.0%)	15 (24.2%)
	3. I prefer solitude and avoid communication with others.	2 (4.9%)	4 (6.5%)
Q4. Anxiety or Worry Related to Diagnosis	0. I feel calm and relaxed most of the time.	6 (14.6%)	7 (11.3%)
	1. I occasionally feel anxious, but it’s manageable.	15 (36.6%)	26 (41.9%)
	2. I frequently feel anxious and find it difficult to control.	16 (39.0%)	24 (38.7%)
	3. I constantly feel anxious and overwhelmed.	4 (9.8%)	5 (8.1%)

**Table 7:** Spearman’s correlations between emotional responses to heart disease diagnosis.

Variable		Q1	Q2	Q3	Q4
1. Q1	Spearman’s rho	—			
	*p*-value	—			
2. Q2	Spearman’s rho	0.591	—		
	*p*-value	<0.001	—		
3. Q3	Spearman’s rho	0.654	0.517	—	
	*p*-value	<0.001	<0.001	—	
4. Q4	Spearman’s rho	0.542	0.562	0.648	—
	*p*-value	<0.001	<0.001	<0.001	—

**Table 8:** Impact of heart disease on daily life and functional capacity.

		Female 41 Patients	Male 62 Patients
Subsection 4: Daily Life and Functional Impact			
Variable	Level	Counts	Counts
Q5. Work Performance or Motivation Changes	0. My work performance is consistent with pre-diagnosis levels.	4 (9.8%)	17 (27.4%)
	1. I need to put in extra effort to fulfill responsibilities, but I manage.	19 (46.3%)	24 (38.7%)
	2. I struggle to motivate myself to work at full capacity.	13 (31.7%)	14 (22.6%)
	3. I find it very difficult to fulfill my work responsibilities.	5 (12.2%)	7 (11.3%)
Q6. Physical Symptoms and Daily Activities	0. I can perform daily activities without any physical issues.	4 (9.8%)	9 (14.5%)
	1. I experience minor physical discomfort but can manage most activities.	23 (56.1%)	28 (45.2%)
	2. Physical symptoms frequently interfere with my ability to perform tasks.	13 (31.7%)	18 (29.0%)
	3. Physical symptoms make it very difficult to carry out daily tasks.	1 (2.4%)	7 (11.3%)

**Table 9:** Spearman’s correlation between work performance changes and physical symptom impact on daily activities.

Variable		Q5	Q6
1. Q5	Spearman’s rho	—	
	*p*-value	—	
2. Q6	Spearman’s rho	0.547	—
	*p*-value	<0.001	—

**Table 10:** Future Outlook, quality of life, and interest in romantic relationships among patients with heart disease.

		Female 41 Patients	Male 62 Patients
Subsection 5: Future Outlook and Coping			
Variable	Level	Counts	Counts
Q7. Future Outlook in Light of Heart Condition	0. I feel optimistic about my future health and well-being.	2 (4.9%)	12 (19.4%)
	1. Although I try to stay positive, I sometimes worry about my future.	25 (61.0%)	33 (53.2%)
	2. I have low expectations regarding my long-term health.	10 (24.4%)	12 (19.4%)
	3. I feel my future is bleak because of this condition.	4 (9.8%)	5 (8.1%)
Q8. Quality of life	0. I feel generally content with my life.	3 (7.3%)	8 (12.9%)
	1. I experience some dissatisfaction but manage to cope.	18 (43.9%)	33 (53.2%)
	2. I feel dissatisfied with my life most of the time.	13 (31.7%)	14 (22.6%)
	3. I feel extremely dissatisfied and struggle to find enjoyment in life.	7 (17.1%)	7 (11.3%)
Q9. Interest in Romantic Relationships or Intimacy	0. My feelings toward romantic interests have not changed.	9 (22.0%)	8 (12.9%)
	1. I am slightly less interested in romantic relationships and intimacy.	16 (39.0%)	32 (51.6%)
	2. I have lost a lot of interest in intimacy.	11 (26.8%)	14 (22.6%)
	3. I have completely lost interest in romantic connections.	5 (12.2%)	8 (12.9%)

**Table 11:** Spearman’s correlation analysis of future outlook, quality of life, and interest in romantic relationships or intimacy.

Variable		Q7	Q8	Q9
1. Q7	Spearman’s rho	—		
	*p*-value	—		
2. Q8	Spearman’s rho	0.539	—	
	*p*-value	<0.001	—	
3. Q9	Spearman’s rho	0.702	0.593	—
	*p*-value	<0.001	<0.001	—

**Table 12:** General well-being and emotional impact of heart disease diagnosis.

		Female 41 Patients	Male 62 Patients
Subsection 6: General Well-Being			
Variable	Level	Counts	Counts
Q10. Sleep Quality Since Diagnosis	0. I sleep as well as I did before my diagnosis.	7 (17.1%)	10 (16.1%)
	1. I have more trouble falling asleep and staying asleep than before.	14 (34.1%)	31 (50.0%)
	2. I frequently wake up early and struggle to fall back asleep.	16 (39.0%)	14 (22.6%)
	3. I wake up early and can’t go back to sleep.	4 (9.8%)	7 (11.3%)
Q11. Changes in Energy Levels or Fatigue	0. I feel as energetic as I did before.	4 (9.8%)	9 (14.5%)
	1. I get tired more quickly but can manage daily activities.	19 (46.3%)	29 (46.8%)
	2. I feel tired even with minimal activity.	14 (34.1%)	14 (22.6%)
	3. I feel so exhausted that I struggle to do anything.	4 (9.8%)	10 (16.1%)
Q12. Appetite Changes Since Diagnosis	0. My appetite has remained the same.	4 (9.8%)	18 (29.0%)
	1. My appetite has decreased somewhat compared to before.	19 (64.3%)	27 (43.5%)
	2. I eat less than I used to.	14 (34.1%)	12 (19.4%)
	3. I have little to no appetite.	4 (9.8%)	5 (8.1%)
Q13. Concerns About Overall Health	0. I feel fine and have no significant worries.	8 (19.5%)	10 (16.1%)
	1. I have some concerns, but they do not constantly affect me.	14 (34.1%)	31 (50.0%)
	2. My health problems often dominate my thoughts and feelings.	15 (36.6%)	15 (24.2%)
	3. I am so worried that it’s hard to focus on anything else.	4 (9.8%)	6 (9.7%)
Q14. Thoughts of Self-Harm or Suicide (This question is sensitive. Please remember your responses are confidential.)	0. I have never thought about harming myself.	23 (56.1%)	41 (66.1%)
	1. I have had these thoughts occasionally, but they are very rare.	10 (24.4%)	9 (14.5%)
	2. I sometimes wish to harm myself.	4 (9.8%)	7 (11.3%)
	3. If given the opportunity, I seriously think about ending my life.	4 (9.8%)	5 (8.1%)

**Table 13:** Spearman’s correlation analysis of general well-being variables in IHD patients.

Variable		Q10	Q11	Q12	Q13	Q14
1. Q10	Spearman’s rho	—				
	*p*-value	—				
2. Q11	Spearman’s rho	0.613	—			
	*p*-value	<0.001	—			
3. Q12	Spearman’s rho	0.690	0.643	—		
	*p*-value	<0.001	<0.001	—		
4. Q13	Spearman’s rho	0.736	0.603	0.715	—	
	*p*-value	<0.001	<0.001	<0.001	—	
5. Q14	Spearman’s rho	0.802	0.714	0.740	0.815	—
	*p*-value	<0.001	<0.001	<0.001	<0.001	—

**Table 14:** Overall scale reliability statistics.

Estimate	Cronbach’s α
Point estimate	0.957
95% CI lower bound	0.943
95% CI upper bound	0.968

**Table 15:** Individual item reliability statistics.

	If Item Dropped
Item	Cronbach’s α
Q1	0.954
Q2	0.955
Q3	0.954
Q4	0.955
Q5	0.955
Q6	0.955
Q7	0.954
Q8	0.953
Q9	0.953
Q10	0.954
Q11	0.954
Q12	0.953
Q13	0.952
Q14	0.952

**Table 16:** Chi-square goodness-of-fit test for factor structure.

	Value	df	*p*
Model	98.069	77	0.053

**Table 17:** Factor loadings and uniqueness scores for DA-IHDQ.

Item	Factor 1	Uniqueness
Q13	0.876	0.233
Q14	0.873	0.238
Q12	0.828	0.315
Q9	0.826	0.318
Q8	0.804	0.354
Q7	0.785	0.384
Q11	0.779	0.393
Q10	0.778	0.395
Q3	0.773	0.403
Q1	0.763	0.418
Q6	0.740	0.453
Q5	0.726	0.473
Q2	0.725	0.475
Q4	0.706	0.501

Note: Applied rotation method is varimax.

**Table 18:** Factor characteristics and variance explained.

		Unrotated Solution			Rotated Solution		
	Eigenvalues	SumSq. Loadings	Proportion Var.	Cumulative	SumSq. Loadings	Proportion Var.	Cumulative
Factor 1	9.021	8.648	0.618	0.618	8.648	0.618	0.618

**Table 19:** Parallel analysis of eigenvalues.

	Real Data Component Eigenvalues	Simulated Data Mean Eigenvalues
Factor 1 *	9.021	1.694
Factor 2	0.709	1.527
Factor 3	0.605	1.375
Factor 4	0.592	1.282
Factor 5	0.484	1.182
Factor 6	0.470	1.085
Factor 7	0.390	0.996
Factor 8	0.337	0.912
Factor 9	0.306	0.838
Factor 10	0.279	0.769
Factor 11	0.249	0.703
Factor 12	0.209	0.626
Factor 13	0.200	0.549
Factor 14	0.149	0.461

Note: ‘*’ = Factor should be retained. Results from PC-based parallel analysis.

**Table 20:** Pearson’s correlation between DA-IHDQ and BDI-II scores.

Variable		DA-IHDQ	BDI-II
DA-IHDQ	Pearson’s r	—	
	*p*-value	—	
BDI-II	Pearson’s r	0.935	—
	*p*-value	<0.001	—

**Table 21:** Confusion matrix interpretation.

	Gold Positive	Gold Negative	Total
Test Positive	18	1	19
Test Negative	2	82	84
Total	20	83	103

**Table 22:** Sample distribution interpretation.

	n
Total	103
Without Depression	20
With Depression	83
Positive Tests	19
Negative Tests	84
True Test	100
Wrong Test	3

**Table 23:** Diagnostic accuracy metrics interpretation.

	Ratios
Sensitivity	90.0%
Specificity	98.8%
Accuracy	97.1%
Prevalence	19.4%
Positive Predictive Value	94.7%
Negative Predictive Value	97.6%
Post-test Without Depression Probability	94.7%
Post-test With Depression Probability	97.6%
Positive Likelihood Ratio	74.7
Negative Likelihood Ratio	0.101

## Data Availability

The raw data supporting the conclusions of this article will be made available by the authors on request.

## Supplementary Materials

The following supporting information can be downloaded at https://www.mdpi.com/article/10.3390/jcm14030837/s1, File S1: Depression assessment in Ischemic Heart Disease Questionnaire (DA-IHDQ), File S2: Psychometric validation of the depression assessment in Ischemic Heart Disease Questionnaire (DA-IHDQ).
