# Supplementary material for: Screening Depression in Ischemic Heart Disease: Gender Differences and Psychosocial Implications Using a Self-Developed Questionnaire
Source: J Clin Med. 2025 Jan 27;14(3):837. doi: 10.3390/jcm14030837 (PMC11818656; doi:10.3390/jcm14030837)
Supplement: Supplementary file 1 [file jcm-14-00837-s001.zip › File S1.pdf]

Depression Assessment in Ischemic Heart Disease Questionnaire (DA-IHDQ)

Instructions:

This questionnaire aims to assess the emotional and psychological experiences of patients with ischemic heart disease. Your responses will help identify factors related to mood, well-being, and coping mechanisms.

Please read each question carefully and select the option that best describes your feelings and experiences. There are no right or wrong answers. Your responses will remain confidential and will be used for statistical purposes only.

Section I: Demographic and Clinical Data:

Subsection 1: Demographic Information

1. **Gender:**
   - o Female
   - o Male

2. **Age:**
   - o < 40
   - o 40-59
   - o 60-79
   - o 80-89

3. **Environment:**
   - o Urban
   - o Rural

4. **Marital Status:**
   - o Married
   - o Single
   - o Widowed
   - o Divorced

5. **Social Status:**
   - o Employed
   - o Disability Pension
   - o Unemployed
   - o Age Pension

Subsection 2: Medical History of Heart Disease

6. **Type of Ischemic Heart Disease:**
   - o Unstable Angina Pectoris
   - o Acute Myocardial Infarction
   - o Stable Angina Pectoris

- Silent Myocardial Infarction
- Other: ______

7. **Onset of Ischemic Heart Disease:**
    - < 1 month
    - 1-3 months
    - 3-6 months
    - 6-12 months
    - 1-3 years
    - > 3 years

8. **Risk Factors (select all that apply):**
    - Hypertension
    - Smoking
    - Alcohol
    - Obesity
    - Diabetes
    - Hypercholesterolemia
    - Hypertriglyceridemia
    - Inflammation
    - Tachycardia
    - Genetic factors
    - Other: ______

Section II: Psychological/Psychiatric and Physical Symptoms:

Subsection 3: Emotional Responses to Diagnosis

1. **Feelings about Heart Disease Diagnosis:**
    - I feel positive and confident about managing my condition.
    - Sometimes I feel a bit sad about my diagnosis.
    - Occasionally, I feel overwhelmed by sadness, but not all the time.
    - I often feel a deep sadness that I can't seem to overcome.

2. **Changes in Frustration or Anger Since Diagnosis:**
    - I manage my emotions as I did before the diagnosis.
    - I get frustrated more easily than before but rarely feel angry.
    - I get irritated easily, and even small things can trigger my anger.
    - I feel constantly angry because of my health issues.

3. **Changes in Communication Since Diagnosis:**
    - I communicate as well as I did before my diagnosis.
    - I find it slightly harder to express my feelings but still try to communicate.
    - I feel indifferent to social interactions and somewhat detached.
    - I prefer solitude and avoid communication with others.

4. **Anxiety or Worry Related to Diagnosis:**
   - I feel calm and relaxed most of the time.
   - I occasionally feel anxious, but it's manageable.
   - I frequently feel anxious and find it difficult to control.
   - I constantly feel anxious and overwhelmed.

Subsection 4: Daily Life and Functional Impact

5. **Work Performance or Motivation Changes:**
   - My work performance is consistent with pre-diagnosis levels.
   - I need to put in extra effort to fulfill responsibilities, but I manage.
   - I struggle to motivate myself to work at full capacity.
   - I find it very difficult to fulfill my work responsibilities.

6. **Physical Symptoms and Daily Activities:**
   - I can perform daily activities without any physical issues.
   - I experience minor physical discomfort but can manage most activities.
   - Physical symptoms frequently interfere with my ability to perform tasks.
   - Physical symptoms make it very difficult to carry out daily tasks.

Subsection 5: Future Outlook and Coping

7. **Future Outlook in Light of Heart Condition:**
   - I feel optimistic about my future health and well-being.
   - Although I try to stay positive, I sometimes worry about my future.
   - I have low expectations regarding my long-term health.
   - I feel my future is bleak because of this condition.

8. **Quality of life:**
   - I feel generally content with my life.
   - I experience some dissatisfaction but manage to cope.
   - I feel dissatisfied with my life most of the time.
   - I feel extremely dissatisfied and struggle to find enjoyment in life.

9. **Interest in Romantic Relationships or Intimacy:**
   - My feelings toward romantic interests have not changed.
   - I am slightly less interested in romantic relationships and intimacy.
   - I have lost a lot of interest in intimacy.
   - I have completely lost interest in romantic connections.

Subsection 6: General Well-Being

10. **Sleep Quality Since Diagnosis:**
    - I sleep as well as I did before my diagnosis.
    - I have more trouble falling asleep and staying asleep than before.
    - I frequently wake up early and struggle to fall back asleep.
    - I wake up early and can't go back to sleep.

11. **Changes in Energy Levels or Fatigue:**
    - I feel as energetic as I did before.
    - I get tired more quickly but can manage daily activities.
    - I feel tired even with minimal activity.
    - I feel so exhausted that I struggle to do anything.

12. **Appetite Changes Since Diagnosis:**
    - My appetite has remained the same.
    - My appetite has decreased somewhat compared to before.
    - I eat less than I used to.
    - I have little to no appetite.

13. **Concerns About Overall Health:**
    - I feel fine and have no significant worries.
    - I have some concerns, but they do not constantly affect me.
    - My health problems often dominate my thoughts and feelings.
    - I am so worried that it's hard to focus on anything else.

14. **Thoughts of Self-Harm or Suicide:**
    (This question is sensitive. Please remember your responses are confidential.)
    - I have never thought about harming myself.
    - I have had these thoughts occasionally, but they are very rare.
    - I sometimes wish to harm myself.
    - If given the opportunity, I seriously think about ending my life.

Supplementary Section:

1. **Interest in Receiving Psychological/Psychiatric Help:**
    - Yes
    - No
