# Supplementary material for: Screening Depression in Ischemic Heart Disease: Gender Differences and Psychosocial Implications Using a Self-Developed Questionnaire
Source: J Clin Med. 2025 Jan 27;14(3):837. doi: 10.3390/jcm14030837 (PMC11818656; doi:10.3390/jcm14030837)
Supplement: Supplementary file 1 [file jcm-14-00837-s001.zip › File S2.pdf]

Psychometric Validation of the Depression Assessment in Ischemic Heart Disease Questionnaire (DA-IHDQ)

1. Questionnaire Development Process

The DA-IHDQ was developed as a targeted screening tool for detecting depression severity in patients with ischemic heart disease. It consists of 23 items divided into two sections:

- Demographic and Clinical Data: Gender, age, marital status, social status, and medical history.
- Psychological/Psychiatric and Physical Symptoms: 14 items rated on a 0–3 Likert scale, with total scores ranging from 0 to 42, classified as:
  - 0–10: Minimal to no depression
  - 11–20: Mild Depression
  - 21–30: Moderate Depression
  - 31–42: Severe Depression

2. Reliability Testing

- Internal consistency: Cronbach's α = 0.957, indicating excellent reliability.
- Item-total correlations: Each item contributed meaningfully; removing any item did not significantly change α (range: 0.943–0.968).

3. Construct Validity

Exploratory Factor Analysis using Principal Component Analysis with Varimax rotation suggested a one-factor model, explaining 61.8% of the total variance.

- Goodness-of-fit test: $\chi^2 = 98.069$, $p = 0.053$ → acceptable model fit.
- Parallel analysis: The first factor's eigenvalue (9.021) exceeded the simulated threshold (1.694), while the second factor (0.709) was below its expected cutoff (1.527), confirming a unidimensional structure.
- Factor loadings (range: 0.706–0.876):
  - Highest loadings: Q13 (0.876), Q14 (0.873), Q12 (0.828) → Reflect health concerns, emotional distress, and psychological responses.
    Lowest loading: Q4 (0.706) → Still exceeds the 0.40 threshold, confirming its relevance.

4. Criterion Validity

The DA-IHDQ demonstrated strong validity by correlating highly with standardized depression measures:

- Beck Depression Inventory-II (BDI-II): $r = 0.935$, $p < .001$

This confirms the DA-IHDQ effectively measures depression severity in IHD patients.

5. Sensitivity and Specificity

The DA-IHDQ showed high diagnostic accuracy, correctly classifying 97.1% of cases.

- Sensitivity: 90.0% (correctly identified depressed individuals)
- Specificity: 98.8% (minimized false positives)
- Positive Predictive Value: 94.7%
- Negative Predictive Value: 97.6%
- Positive Likelihood Ratio: 74.7 (a positive result strongly increases depression probability)
- Negative Likelihood Ratio: 0.101 (a negative result effectively reduces depression likelihood)

The test correctly classified 18/20 depressed cases and 82/83 non-depressed cases, with only 3 misclassifications in a sample of 103 participants.

6. Conclusion

The DA-IHDQ demonstrates robust psychometric properties, including high internal consistency, strong criterion validity, and excellent diagnostic accuracy. The high sensitivity and specificity indicate its potential as a reliable depression screening tool for IHD patients. Future validation in larger, multi-center cohorts is recommended to confirm its applicability across diverse populations and clinical settings.
